# Arthroscopic-assisted uniportal spinal surgery with bone-anchored annular repair for far lateral lumbar disc herniation: A case report

**DOI:** 10.1097/MD.0000000000051107

**Published:** 2026-10-16

**Authors:** Hao Cao, Xin Zhang, Shili Chen, Dong Liu, Fei Sun, En Song

**Affiliations:** a Orthopedics, Kunming Orthopedic Hospital, Kunming, Yunnan, China; b Department of Orthopedics and Traumatology, Luliang County Traditional Chinese Medicine Hospital, Qujing, Yunnan, China; c Spinal Department, The First Affiliated Hospital of Kunming Medical University, Kunming, Yunnan, China.

**Keywords:** annular repair, arthroscopic-assisted uniportal spinal surgery, far lateral lumbar disc herniation

## Abstract

**Rationale:**

Far lateral lumbar disc herniation (FLLDH) is technically challenging because of its foraminal or extraforaminal location. Arthroscopic-assisted uniportal spinal surgery (AUSS) with bone-anchored annular repair has previously been described for lumbar disc herniation, but its use through an extraforaminal corridor for FLLDH has not been specifically detailed.

**Patient concerns:**

A 50-year-old woman presented with a 4-month history of refractory lumbosacral and gluteal pain, followed by acute right lower-extremity radicular pain along the L3–L4 dermatome. Walking was limited to <20 minutes. Her leg-pain visual analog scale (VAS) score was 7, and her Oswestry Disability Index (ODI) was 45/50 (90%) despite conservative treatment.

**Diagnoses:**

Computed tomography and magnetic resonance imaging demonstrated a right-sided far-lateral L3–L4 disc herniation with foraminal stenosis and compression of the exiting L3 nerve root. Dynamic radiographs showed no segmental instability.

**Interventions:**

The patient underwent AUSS-assisted far lateral discectomy under general anesthesia through a 1.5 to 2.0-cm extraforaminal incision. Foraminal decompression, removal of the herniated nucleus pulposus, and bone-anchored annular repair were performed through the same uniportal approach.

**Outcomes:**

The operation lasted 45 minutes, with an estimated blood loss of approximately 10 mL. No perioperative complications were documented. The leg-pain VAS score decreased from 7 to 1, and the ODI decreased from 45/50 (90%) to 10/50 (20%). Postoperative day 1 imaging confirmed removal of the herniated fragment and adequate decompression of the exiting L3 nerve root. At the 15-month telephone-only follow-up, the patient-reported leg-pain VAS score was 0 and the ODI was 8/50 (16%); no physical examination or interval imaging was obtained.

**Lessons:**

This case demonstrates the technical feasibility of performing decompression and bone-anchored annular repair for FLLDH through an extraforaminal AUSS corridor. The contribution is the anatomical and procedural application of a previously described repair concept rather than the introduction of a new repair technique. Because the 15-month follow-up was telephone-based and lacked neurological examination and interval imaging, this report does not establish structural healing, recurrence prevention, long-term safety, comparative effectiveness, or superiority.

## 1. Introduction

Approximately 10% of symptomatic lumbar disc herniations are located in or lateral to the neural foramen. These intraforaminal and extraforaminal lesions, commonly termed far-lateral lumbar disc herniations (FLLDH), compress the exiting nerve root and dorsal root ganglion and may cause severe radicular pain that is resistant to conservative treatment. Their location creates diagnostic and operative challenges. Traditional open approaches may require substantial paraspinal muscle dissection and, depending on the exposure, removal of bone near the facet joint, with potential blood loss and postoperative instability.^[1,2]^ Spinal endoscopy can provide a smaller access corridor and direct visualization. Unilateral biportal endoscopy (UBE) has been used for lumbar decompression,^[3–6]^ but it has a recognized learning curve and may present challenges in orientation, instrument control, and maintenance of the visual field.^[7,8]^

UBE uses separate viewing and working portals, allowing triangulation and the use of conventional decompression instruments.^[5,9]^ Reported technical challenges include instrument control, orientation, and intermittent visual-field obstruction, particularly during the learning phase.^[7,8]^ assisted uniportal spinal surgery (AUSS), also termed uniportal non-coaxial endoscopic spine surgery, places the viewing and working channels within a single incision.^[10–12]^ This configuration permits non-coaxial instrument manipulation through one portal, but any comparative advantages in operative time, blood loss, recovery, or clinical outcome require direct evaluation. AUSS has also been evaluated for FLLDH in a retrospective comparison with percutaneous endoscopic lumbar discectomy.^[13]^ However, that study focused on decompression and did not specifically describe bone-anchored annular repair through the same extraforaminal corridor.

Our group previously reported AUSS with ligamentum-flavum preservation and bone-anchored annular suturing for L5–S1 lumbar disc herniation through a posterior approach.^[12]^ That report described the bone-anchored repair concept but did not address far-lateral or extraforaminal pathology. In the present case, the previously described repair concept was applied to a right L3–L4 FLLDH through an extraforaminal AUSS corridor, with decompression of the exiting L3 nerve root and annular repair performed through the same uniportal incision. Accordingly, the novelty of this report lies in its anatomical and procedural application to FLLDH rather than in the invention of bone-anchored annular suturing itself. The purpose of this technical case report is to describe this operative workflow and its technical feasibility. A single case cannot establish clinical superiority, comparative safety, long-term structural safety, or effectiveness.

## 2. Case description

A 50-year-old woman presented with a 4-month history of refractory chronic lumbosacral and gluteal pain, which progressed to acute right lower-extremity radiculopathy. The neuropathic pain radiated along the right L3–L4 dermatome, extending from the hip to the anterolateral thigh, resulting in significant gait impairment with functional ambulation limited to <20 minutes per episode. Upon admission, the patient’s visual analog scale (VAS) score for radiating leg pain was 7, and her Oswestry Disability Index (ODI) was 45. The patient had no prior medical history of hypertension, diabetes, or coronary heart disease, no history of spinal surgery, and no family history of hereditary diseases. Prior to her visit, the patient had been taking oral anti-inflammatory and analgesic medications for over 4 months, but her symptoms showed no significant improvement. Neurological examination demonstrated preserved motor strength (Grade V bilaterally), intact sensation, normal sphincter function, a positive straight leg raise test at 70° on the right, normoreflexia in both lower limbs, and no pathological reflexes (Babinski sign negative). Dynamic lumbar radiographs (flexion-extension views) confirmed segmental stability without pathological motion, while computed tomography (CT) revealed multilevel lumbar disc herniations (L2–L3, L3–L4, L4–L5) with right-sided foraminal stenosis at L3–L4. Subsequent magnetic resonance imaging (MRI) corroborated these findings, demonstrating a right-sided far-lateral disc herniation at L3–L4 with concomitant foraminal stenosis (Fig. 1). Based on the patient’s medical history, physical examination, and imaging results, the primary diagnosis was a right-sided far-lateral L3–L4 disc herniation. Lumbar spinal stenosis was ruled out because the patient primarily presented with radicular pain rather than typical intermittent claudication, and imaging did not reveal significant central spinal canal narrowing. After conservative treatment failed, surgery was selected to decompress the exiting L3 nerve root. AUSS-assisted far-lateral discectomy with annular repair was planned after discussion of the operative options, expected benefits, and potential risks.

**Figure 1.**
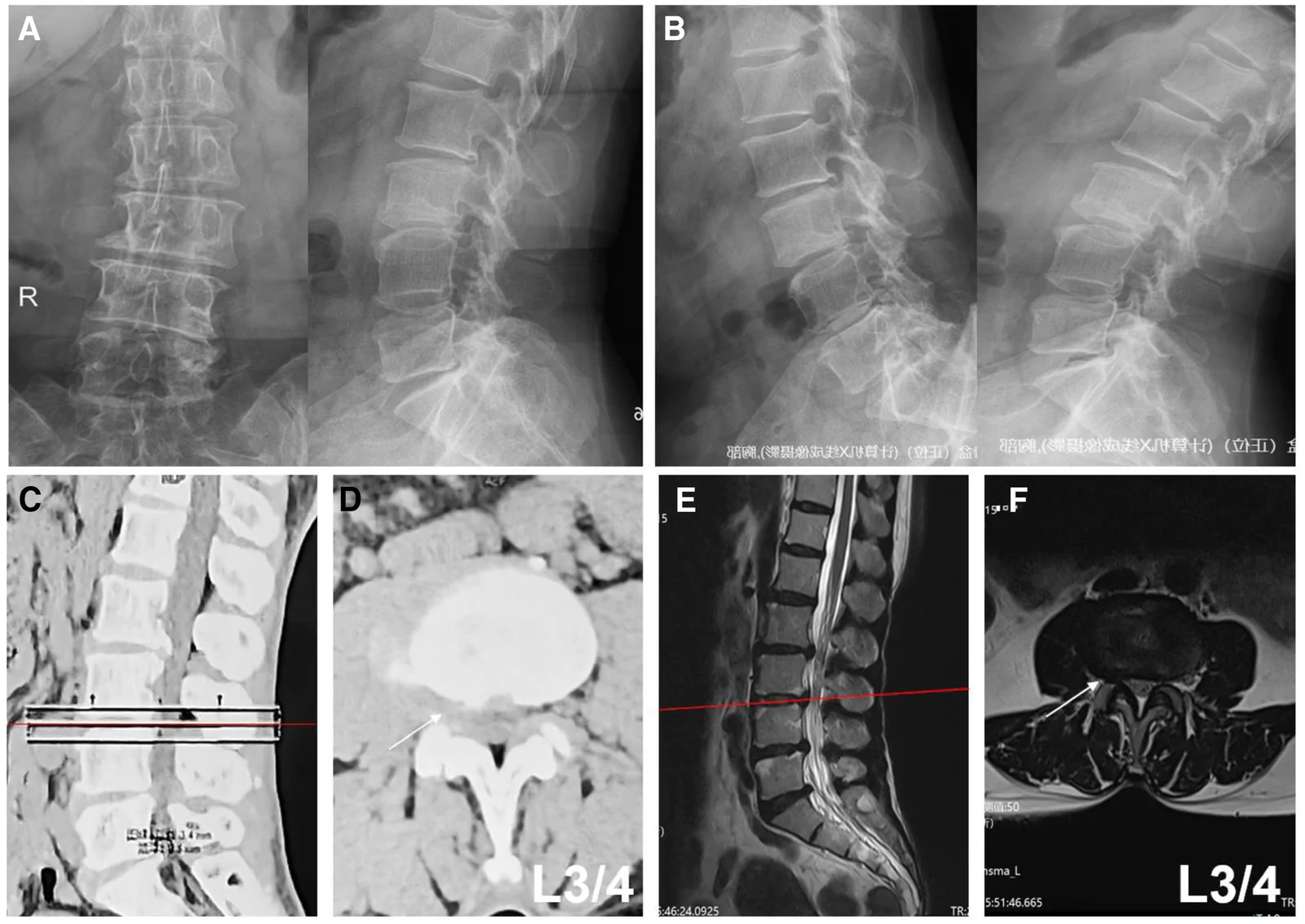
Preoperative imaging examination of the lumbar spine. (A) Anteroposterior and lateral X-rays of the lumbar spine and (B) dynamic X-rays of the lumbar spine demonstrate the relative dynamic stability of all lumbar segments. Sagittal (C) and axial (D) CT scans of the lumbar spine reveal L3–L4 disc herniation and right foraminal stenosis. Sagittal (E) and axial (F) MRI scans of the lumbar spine show a right lateral disc herniation accompanied by foraminal stenosis. The white arrows indicate the herniated disc. CT = computed tomography, MRI = magnetic resonance imaging.

The patient provided written informed consent for the surgical procedure and for publication of the case details and accompanying images. This case report was approved by the Ethics Committee of The First Affiliated Hospital of Kunming Medical University (No. L171; July 16, 2024).

### 2.1. Surgical procedure

#### 2.1.1. Positioning, incision, and instrumentation

The procedure was conducted under general endotracheal anesthesia with the patient positioned in a standardized prone posture. Optimal cervical alignment was maintained using a horseshoe-shaped headrest with slight flexion. Following sterile preparation and draping, intraoperative C-arm fluoroscopy was employed to accurately localize the target L3–L4 spinal segment. A 2-cm longitudinal skin incision was then carefully made, oriented parallel to the L3–L4 disc space and centered over the anatomical landmark of the L3 transverse process tip (Fig. 2).

**Figure 2.**
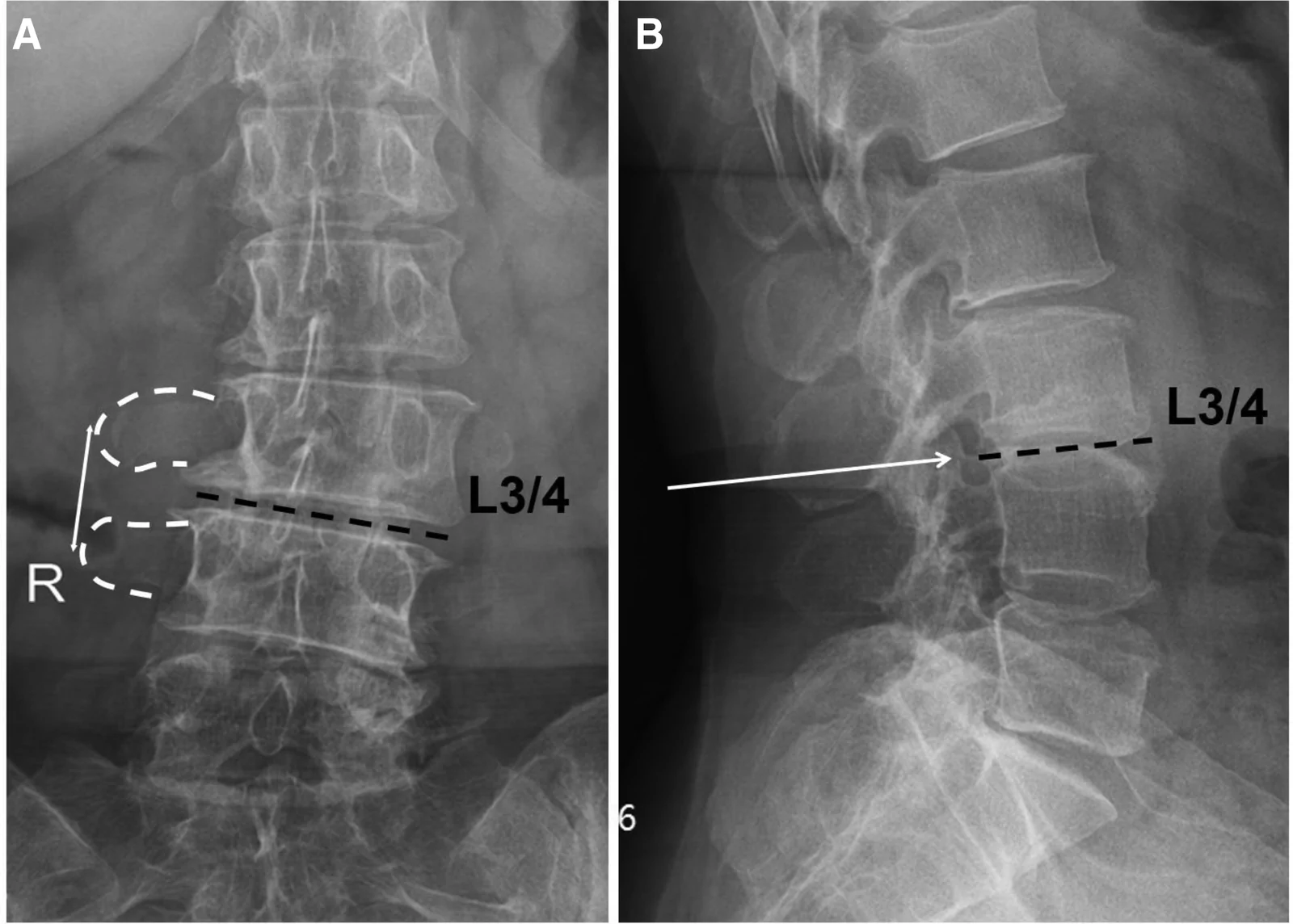
X-ray schematic of the surgical approach. (A) Preoperative positioning schematic on the lumbar anteroposterior X-ray. The tip of the L3 transverse process is selected as the upper reference point, and the tip of the L4 transverse process as the lower reference point. The white arrow indicates the reference line for the incision, while the white dashed lines outline the right transverse processes of L3 and L4. The black dashed line marks the L3–L4 intervertebral space. (B) Preoperative positioning schematic on the lumbar lateral X-ray. The white arrow indicates the direction for establishing the surgical pathway, and the black dashed line marks the L3–L4 intervertebral space.

#### 2.1.2. AUSS extraforaminal approach

Incision Placement: The longitudinal surgical incision is approximately 1.5 to 2.0 cm in length and is positioned 2 to 3 cm lateral to the isthmus (Fig. 3).

**Figure 3.**
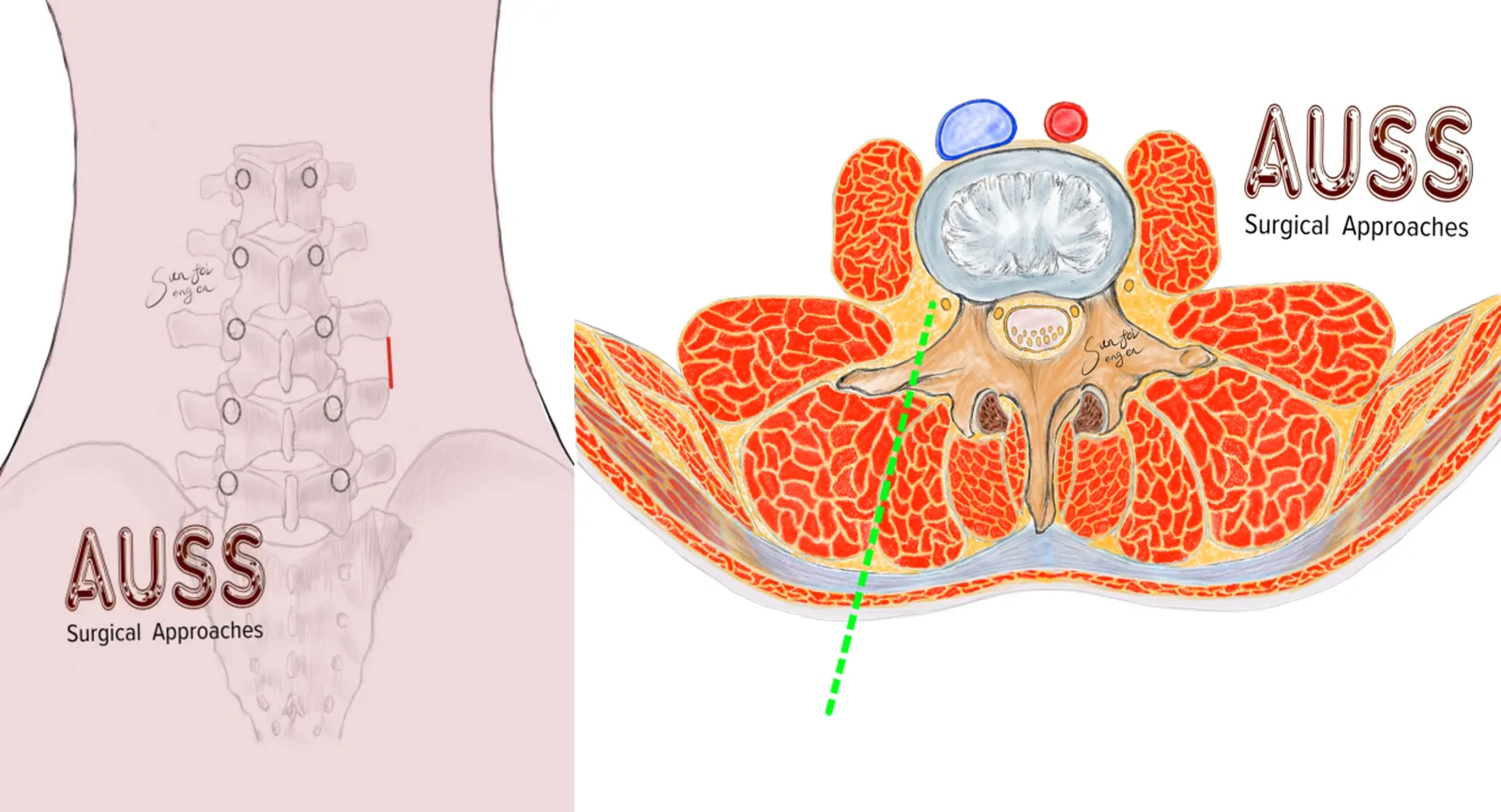
Schematic diagram of the AUSS far-lateral (extraforaminal) approach. AUSS = arthroscopic-assisted uniportal spinal surgery.

Indications: Far lateral disc herniation, foraminal stenosis, far lateral syndrome, Bertolotti syndrome, and transforaminal lumbar interbody fusion procedures.

Direct localization (rapid localization) method: A longitudinal incision is made along the line connecting the tips of the transverse processes of the adjacent vertebrae, oriented laterally toward the foramen region.

#### 2.1.3. AUSS-assisted far lateral discectomy

Initially, a serial dilation technique was performed through the incision using tissue expanders to sequentially dilate the subcutaneous tissue and lumbodorsal muscular fascia. Complete soft tissue clearance from the lamina surface was then carried out using a dissector. The arthroscope (Arthrex Synergy, Shanghai, P.R. China) was introduced and connected to a continuous gravity-fed saline irrigation system. Under direct visualization, radiofrequency ablation (Anhui Backbone Biotechnology Co., Ltd., Hefei, Anhui, P.R. China) was used to carefully remove the soft tissues covering the inferior articular process of L3, the superior articular process of L4, and the L3–L4 intervertebral space, thereby exposing the foraminal region. A 4-mm high-speed burr (Zirui Technology, Guizhou, China) was utilized to partially resect the pars interarticularis, revealing the tip of the superior articular process and the articular cartilage of L4. Foraminal decompression was subsequently performed using a 2-mm 130° angled punch (Anhui Backbone Biotechnology Co., Ltd.) and further bony removal. After resection of the ligamentum flavum, neurovascular structures – including adipose tissue and the vascular plexus – were carefully dissected with meticulous hemostasis, fully exposing the L3 exiting nerve root and herniated disc. Finally, the protruded nucleus pulposus was completely removed using pituitary rongeurs, achieving complete decompression of the L3 exiting nerve root (Fig. 4).

**Figure 4.**
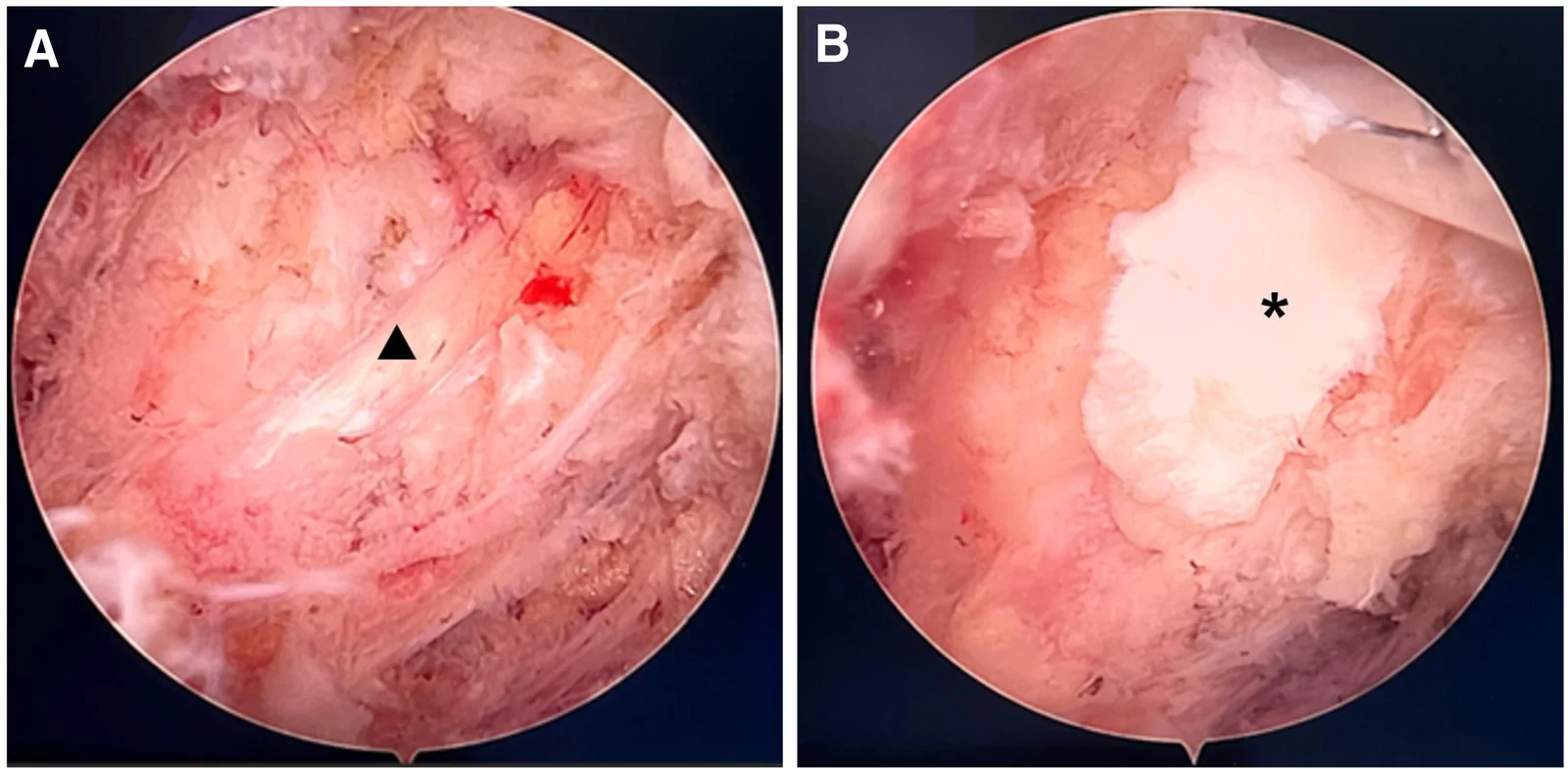
Arthroscopic decompression and discectomy. (A) Radiofrequency ablation is used to expose the exiting L3 nerve root and intervertebral disc. (B) The herniated nucleus pulposus is removed with pituitary rongeurs. ▲, exiting L3 nerve root; *, nucleus pulposus.

#### 2.1.4. AUSS-assisted annular repair through the extraforaminal corridor

The bone-anchored annular repair concept followed the method previously described by Xia et al^[12]^; this case does not claim a newly developed repair device or knot-advancement principle. The procedural adaptation was performing the repair through the far-lateral extraforaminal AUSS corridor after decompression of the exiting L3 nerve root. Following removal of the herniated nucleus pulposus, direct visualization revealed a well-demarcated annular tear at the vertebral margin. The defect was repaired using an annular repair device (2020 Medical Technology Company, Beijing, China). The white suture-loop device was placed 2 mm medial to the tear margin (spinal canal aspect), and the suture carrier was advanced through the annular fibers and withdrawn. The green suture was then threaded through the deployed white loop and introduced 2 mm lateral to the defect (foraminal aspect). Engagement was confirmed by visualizing the green suture within the white loop after carrier withdrawal. The white suture was tensioned to form the initial knot, and a dedicated knot pusher was used to advance and tighten the white and green sutures. Residual suture material was trimmed. At the end of the procedure, the tear margins appeared approximated under arthroscopic visualization (Fig. 5); biomechanical strength and long-term healing were not assessed in this case.

**Figure 5.**
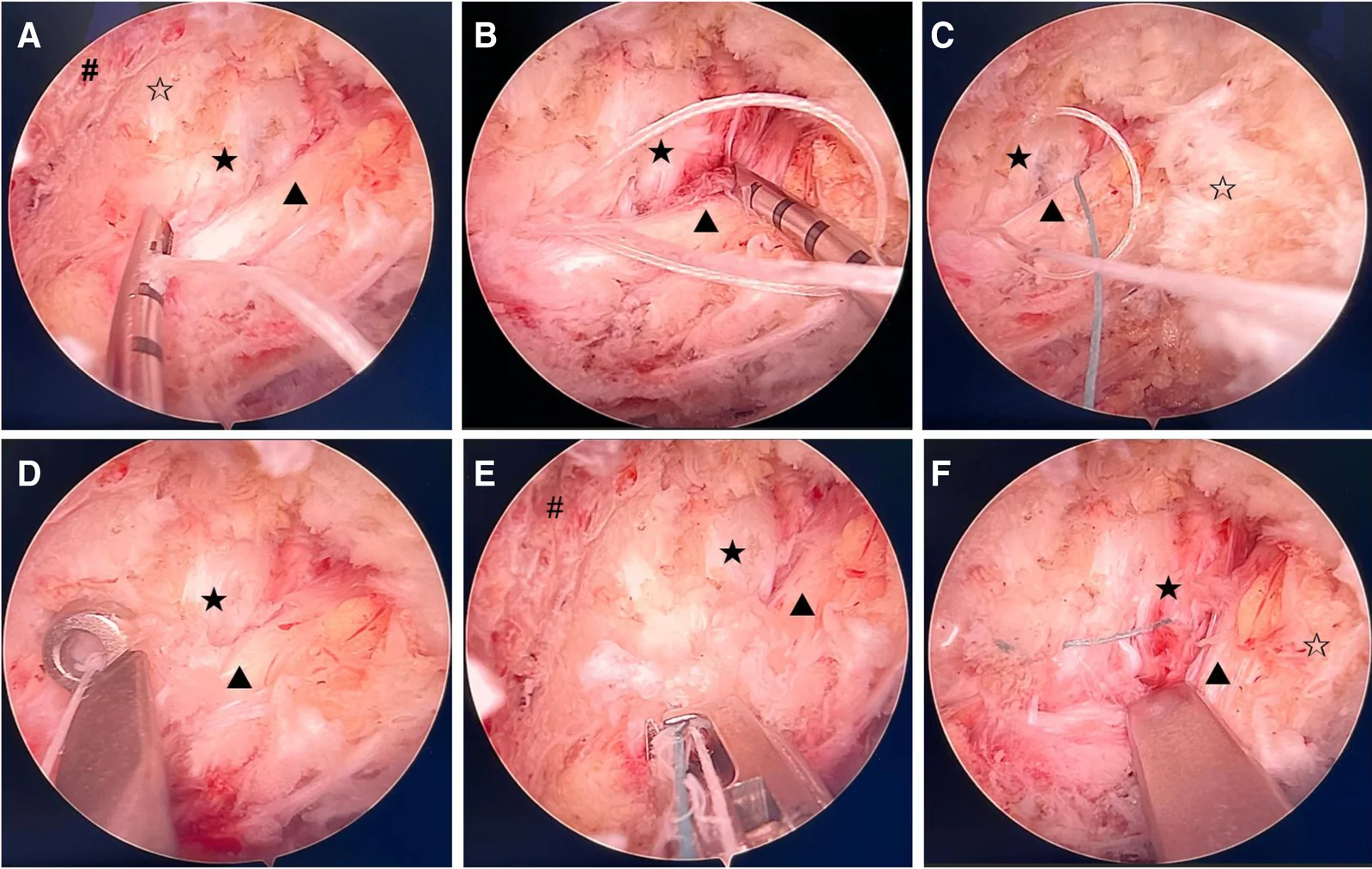
Arthroscopic bone-anchored annular repair through the extraforaminal AUSS corridor. (A) A white looped suture is passed through the medial margin of the annular defect. (B) The suture carrier is withdrawn, leaving the loop in place. (C) A green suture is passed through the white loop and subsequently through the lateral margin of the defect. (D) Tensioning the white suture forms the initial knot. (E) A knot pusher is used to advance and tighten the suture construct. (F) The excess suture is trimmed, and approximation of the annular defect margins is confirmed under arthroscopic visualization. ▲, exiting L3 nerve root; ★, annulus fibrosus; ☆, ligamentum flavum; #, superior articular process of L4. AUSS = arthroscopic-assisted uniportal spinal surgery.

#### 2.1.5. Drainage and closure

A closed suction drainage tube was aseptically placed prior to wound closure. The surgical incision was meticulously repaired in layers using interrupted silk sutures. Operative records indicate a blood loss of 10 mL and a total surgical duration of 45 minutes.

### 2.2. Postoperative outcomes, rehabilitation, and follow-up

Immediately after surgery, the patient reported marked improvement in right lower-extremity and gluteal pain. The leg-pain VAS score decreased from 7 preoperatively to 1 postoperatively, and the ODI decreased from 45/50 (90%) to 10/50 (20%). The drain was removed at 48 hours, after which the patient began ambulation as instructed. Postoperative CT and MRI confirmed removal of the far-lateral herniated fragment and adequate decompression of the exiting L3 nerve root (Fig. 6). No documented adverse or unexpected event occurred during the perioperative period, including infection, hemorrhage, new or worsened neurological deficit, cerebrospinal fluid leakage, deep-vein thrombosis, or pulmonary embolism. At the 15-month telephone-only follow-up, the patient reported resolution of right lower-extremity pain, with a leg-pain VAS score of 0 and an ODI of 8/50 (16%), and reported being highly satisfied with the outcome. This assessment was limited to patient-reported symptoms and disability; no neurological examination, radiographs, CT, or MRI was obtained. Consequently, it could not assess annular healing, anchor position or integrity, recurrent disc herniation, or segmental stability. The absence of patient-reported symptoms should not be interpreted as evidence of long-term structural safety. Continued clinical and radiographic follow-up was advised. A specific return-to-work date, detailed activity-progression milestones, the Macnab grade, and EQ-5D data were not prospectively recorded. The clinical timeline is shown in Figure 7.

**Figure 6.**
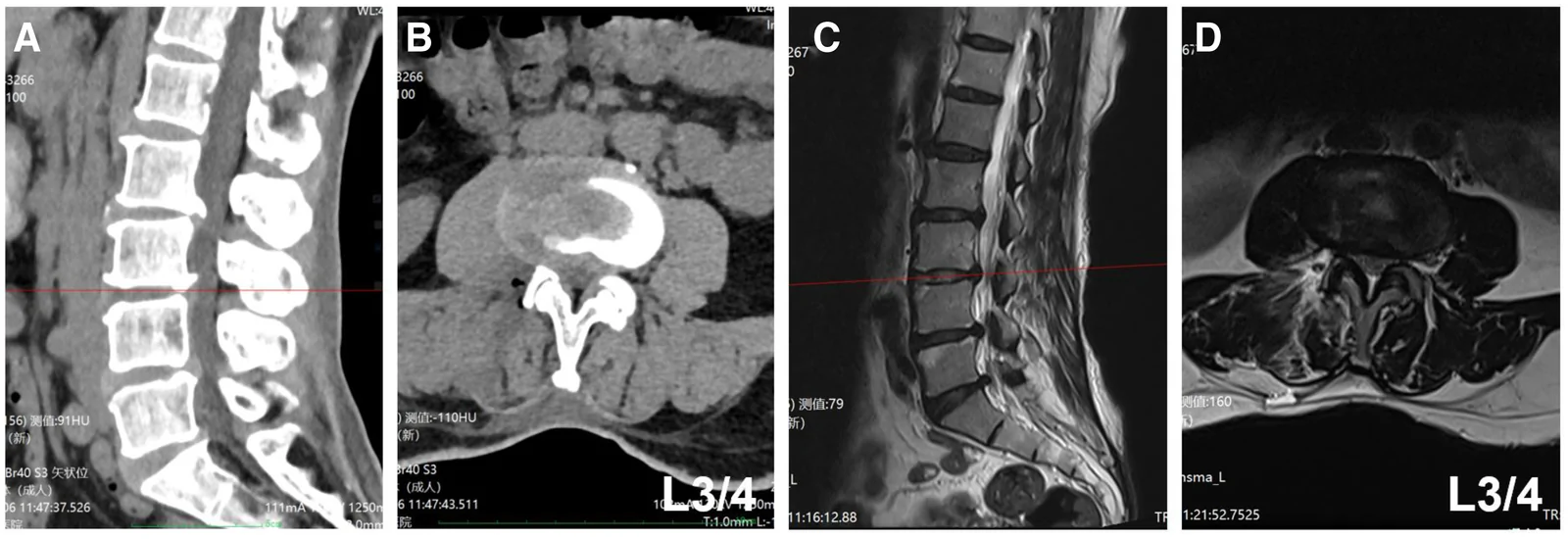
Postoperative imaging of the lumbar spine. On postoperative day 1, sagittal (A) and axial (B) CT images and sagittal (C) and axial (D) MRI images show removal of the right far-lateral L3–L4 disc fragment and decompression of the exiting nerve root.

**Figure 7.**
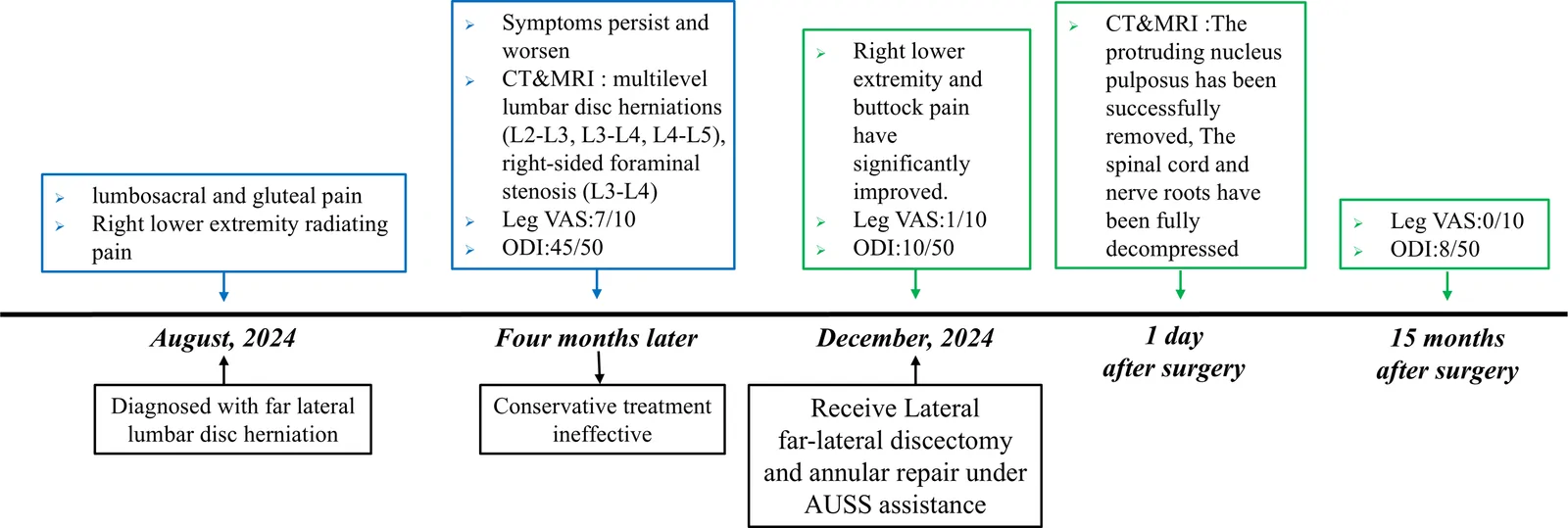
Case follow-up timeline. The timeline distinguishes the preoperative evaluation and intervention, the immediate postoperative clinical and imaging assessment, and the 15-mo telephone-only assessment of patient-reported symptoms and disability. No interval imaging was performed at 15 mo.

## 3. Discussion

The vast majority (>90%) of lumbar disc herniations occur at the posterior disc margin within the spinal canal, presenting as central or paracentral/posterolateral subtypes. In contrast, FLLDH has a distinct foraminal or extraforaminal location and frequently compresses the exiting nerve root.^[14]^ This anatomical pattern can produce severe radiculopathy and requires a specialized operative strategy. AUSS, also termed uniportal non-coaxial spinal endoscopic surgery, permits non-coaxial bimanual manipulation through a single incision. Disruption of the annulus fibrosus contributes to abnormal load distribution and may influence disc degeneration and recurrent herniation,^[15,16]^ while extracellular-matrix degradation and neuroinflammatory mechanisms contribute to nerve-root pathology.^[17]^ Annular repair therefore has a theoretical structural rationale, but its effect on healing, recurrence, or long-term disease progression cannot be determined from this case.

Endoscopic spine surgery has expanded from discectomy to more complex decompression and fusion procedures.^[18,19]^ UBE has broadened minimally invasive options for lumbar disease, while AUSS consolidates the viewing and working channels within a single portal.^[11]^ The technical contribution of this case should be interpreted relative to previous work. Xia et al^[12]^ described posterior ligamentum-flavum-preserving AUSS with bone-anchored annular repair for L5–S1 lumbar disc herniation; the current case applies that previously described repair concept to an L3–L4 far-lateral/extraforaminal lesion, where the working corridor and the neural structure at risk – the exiting L3 root – differ. Thus, the new element is the anatomical and procedural application to FLLDH rather than the underlying repair concept. The observed symptomatic and functional improvements are hypothesis-generating and do not establish biomechanical restoration, recurrence prevention, or segmental stability.

Several established minimally invasive options are available for FLLDH, and each has a different technical profile. AUSS has been evaluated for FLLDH in a retrospective comparison with percutaneous endoscopic lumbar discectomy,^[13]^ but that study did not specifically describe bone-anchored annular repair through the extraforaminal corridor. Percutaneous endoscopic lumbar discectomy provides a small access corridor and direct transforaminal or extraforaminal access, although the constrained working trajectory may limit instrument triangulation and annular suturing in some anatomical settings.^[14]^ Unilateral biportal endoscopy provides independent viewing and working portals, broad triangulation, and familiar decompression instruments, although it requires 2 portals and has a recognized learning curve related to orientation, irrigation, and instrument control.^[3–8]^ Microscopic extraforaminal discectomy provides direct visualization and conventional bimanual handling but may require greater muscle dissection or bony exposure, depending on the approach and anatomy. AUSS places the arthroscope and freely manipulated instruments through a single incision and may permit decompression followed by annular suturing without creating a second portal.^[10–13]^ These are technical distinctions rather than evidence of superiority. Procedure selection should remain individualized according to disc location, foraminal anatomy, surgeon experience, and available equipment.

Annular repair introduces potential risks beyond decompression alone, including suture or anchor loosening, implant migration or failure, local irritation or injury to adjacent neural or osseous structures, infection, recurrent disc herniation, and incomplete healing. Although no perioperative adverse event was documented in this patient, the available follow-up could not evaluate delayed structural or repair-related complications. Therefore, the absence of reported symptoms or complications should not be interpreted as evidence of long-term safety.

Adoption of this combined technique may require experience with uniportal non-coaxial arthroscopic orientation, bimanual instrument control, hemostasis under continuous irrigation, and arthroscopic knot advancement near the exiting nerve root. In addition to standard spinal decompression instruments, the procedure requires an arthroscopic imaging and irrigation platform, radiofrequency and burr systems, and a dedicated annular repair device. These equipment requirements and the implant may increase direct operative cost. The operative time and estimated blood loss observed in this case should not be interpreted as benchmarks during the learning phase. Reproducibility across surgeons and centers remains unproven and should be assessed using standardized training, clearly defined indications, prospective case series, and comparative cost and outcome data.

The estimated blood loss of 10 mL should be interpreted cautiously in an irrigated endoscopic field, and the observed ODI improvement represents an individual patient-reported outcome rather than a performance benchmark. Overall, this report documents the technical execution of the procedure in one patient. Prospective multicenter studies with standardized clinical and imaging follow-up are required to evaluate safety, reproducibility, cost, and clinical durability.

### 3.1. Limitations

The 15-month follow-up was conducted by telephone without neurological examination or interval radiographs, CT, or MRI. Therefore, it documented patient-reported clinical status only and could not assess structural healing, anchor or repair integrity, recurrent disc herniation, or segmental stability. This report included only one patient and no control group, and outcomes were not collected under a prospective protocol. The Macnab grade, EQ-5D, detailed rehabilitation milestones, and return-to-work timing were unavailable. The learning curve, cost, and reproducibility of the combined technique were also not evaluated. Accordingly, the findings are limited to technical feasibility and the clinical course of a single patient and cannot establish comparative effectiveness or long-term safety.

## 4. Conclusion

This case demonstrates the technical feasibility of performing decompression and bone-anchored annular repair for FLLDH through an extraforaminal AUSS approach. Its procedural contribution is the application of a previously described repair concept to a far-lateral lesion. This single case does not establish comparative effectiveness, recurrence prevention, structural healing, or long-term safety. Prospective studies with standardized clinical and imaging follow-up are required.

## Acknowledgments

We thank the patient for consenting to the publication of this case.

## Author Contributions

**Conceptualization:** Hao Cao, Xin Zhang.

**Data curation:** Dong Liu.

**Funding acquisition:** En Song.

**Methodology:** Hao Cao.

**Resources:** Hao Cao, Xin Zhang, Shili Chen.

**Supervision:** Fei Sun, En Song.

**Writing – original draft:** Hao Cao, Xin Zhang, Shili Chen.

**Writing – review & editing:** Hao Cao, Dong Liu, Fei Sun, En Song.
